# Efficacy of low dose pirfenidone in idiopathic pulmonary fibrosis: real world experience from a tertiary university hospital

**DOI:** 10.1038/s41598-020-77837-x

**Published:** 2020-12-04

**Authors:** Myung Jin Song, Sung Woo Moon, Ji Soo Choi, Sang Hoon Lee, Su Hwan Lee, Kyung Soo Chung, Ji Ye Jung, Young Ae Kang, Moo Suk Park, Young Sam Kim, Joon Chang, Song Yee Kim

**Affiliations:** 1 Division of Pulmonary and Critical Care Medicine, Department of Internal Medicine, Seoul National University College of Medicine, Seoul National University Bundang Hospital, Seongnam, South Korea; 2 Division of Pulmonology, Department of Internal Medicine, Severance Hospital, Yonsei University College of Medicine, 50-1 Yonsei-ro, Seodaemun-gu, Seoul, 03722 Republic of Korea

**Keywords:** Respiratory tract diseases, Outcomes research

## Abstract

Pirfenidone is an antifibrotic agent that has been proven to slow down the progression of idiopathic pulmonary fibrosis (IPF). The aim of this study was to evaluate the efficacy of low-dose pirfenidone (that is, less than 1200 mg/day). We retrospectively reviewed the medical records of patients with IPF. The patients were divided into the following three groups, those who were not treated with pirfenidone (control) and those who were treated with pirfenidone at doses < 1200 mg/day (low-dose group) and ≥ 1200 mg/day (high-dose group). The adjusted mean changes in forced vital capacity (FVC) in 1 year were − 200.7, − 88.4, and − 94.7 mL in the control, low-dose, and high-dose groups (p = 0.021). The FVC declined more significantly in the control group than in the low-dose and high-dose groups. No significant difference in FVC change was observed between the low-dose and high-dose groups. Dyspepsia, anorexia, and nausea were significantly more frequent in the low-dose than in the high-dose group, suggesting that dose reduction is attributed to gastrointestinal tract-related adverse events. Dose reduction may help patients to better control gastrointestinal tract-related adverse events; continuing taking the medication at low doses is also expected to be effective in reducing the FVC decline.

## Introduction

Idiopathic pulmonary fibrosis (IPF), a chronic, progressive, fibrotic interstitial lung disease of unknown cause, is characterized by irreversible loss of lung function due to lung scarring, typically occurring in individuals older than 60 years^1,2^. Although the clinical course is highly variable, progressive decline in pulmonary function until eventual death from respiratory failure or complicating comorbidity is inevitable, and median survival is known to be approximately 3 years^3,4^. Despite a poor outcome and devastating respiratory symptoms, no effective therapeutic approach for improving survival besides lung transplantation was available until the emergence of two novel drugs, pirfenidone and nintedanib, which were approved by the Korean Food and Drug Administration in 2012 and 2016, respectively.

Pirfenidone has been proven to slow down forced vital capacity (FVC) decline and reduce the risk of death by 48% at 1 year in a pooled analysis of data from three independent cohorts^5^. Furthermore, it lowered the risk of respiratory-related hospitalization over the course of 1 year^6^. Pirfenidone at a dose of 40 mg/kg/day was tested in a phase II clinical trial^7^. A phase III clinical trial in a Japanese cohort was designed to test 1800 mg/day pirfenidone, which was lower than that administered in the phase II clinical trial^8^, and 2403 mg/day in the United States, Europe, Australia, and North America^9,10^. Finally, pirfenidone was approved with the full recommended dose of 1800 mg/day in Korea and Japan and 2403 mg/day in Europe and the United States. However, in real clinical practice, considerable number of patients take reduced doses, rather than the recommended dose, due to adverse drug reactions^11,12^. A Japanese study, involving all-case post-marketing surveillance of 1371 patients treated with pirfenidone, reported that the most frequent daily dose was less than 1200 mg/day in approximately 60% of patients^13^.

A Japanese phase III clinical trial evaluated high-dose pirfenidone (1800 mg/day), low-dose (1200 mg/day), and placebo and reported that the low-dose group showed a significant effect on disease progression assessed by FVC decline compared with the placebo group^8^. However, the effects of pirfenidone less than 1200 mg/day dose have not been confirmed.

Therefore, the present study aimed to determine the proportion of patients taking low-dose pirfenidone (less than 1200 mg) and evaluate the efficacy of low-dose pirfenidone on disease progression based on FVC change.

## Results

### Patient characteristics

In total, 234 patients with IPF, with 92 patients not treated with pirfenidone (control group) and 142 patients treated with pirfenidone, were enrolled. The median follow-up period of total study population was 25.6 (interquartile range [IQR], 14.1–34.5) months. The median age was 69.0 (IQR, 64.0–74.8) years, and 74.4% patients were men. Patients treated with pirfenidone were divided into the following two groups: those taking less than 1200 mg/day (low-dose group) and those taking 1200 mg/day or more (high-dose group). The baseline characteristics of the study population are shown in Table 1. The median age was higher in the low-dose group than in the control and high-dose groups (68.0 vs. 72.0 vs. 67.0, control vs. low-dose vs. high-dose group in order, p = 0.001). The proportion of male (69.4% vs. 69.6% vs. 87.3%, control vs. low-dose vs. high-dose group in order, p = 0.023) and body mass index (BMI; 23.3 ± 3.0 vs. 24.3 ± 3.0 vs. 25.4 ± 2.7, control vs. low-dose vs. high-dose group in order, p < 0.001) were higher in the high-dose group than in the control and low-dose groups. Predicted FVC % (83.6 ± 20.0 vs. 77.6 ± 12.9 vs. 76.3 ± 13.1, control vs. low-dose vs. high-dose group in order, p = 0.004) and predicted forced expiratory volume in one second % (FEV_1_; 96.2 ± 22.6 vs. 93.3 ± 16.1 vs. 87.6 ± 12.9, control vs. low-dose vs. high-dose group in order, p = 0.005) were higher in the control group than in the other two groups, whereas the actual measured values of FVC and FEV_1_ were not different among the groups. The severity of IPF assessed using the GAP index also did not show a significant difference among the groups.

**Table 1:** Baseline characteristics according to pirfenidone dose.

	Control (n = 92)	Low-dose (n = 79)	High-dose (n = 63)	P value
Sex (male)	64 (69.4%)	55 (69.6%)	55 (87.3%)	0.023
Age (year), IQR	68.0 (62.5–73.0)	72.0 (66.5–76.0)	67.0 (63.0–73.5)	0.001
BMI (kg/m^2^)	23.3 ± 3.0	24.3 ± 3.0	25.4 ± 2.7	< 0.001
Height (cm)	162.8 ± 7.5	162.1 ± 7.5	165.7 ± 6.1	0.008
Weight (kg)	62.0 ± 10.2	64.2 ± 10.4	69.7 ± 8.7	< 0.001
**Smoking exposure (%)**				< 0.001
Never	7 (7.6%)	30 (38.0%)	13 (20.6%)	
Former	51 (55.4%)	35 (44.3%)	45 (71.4%)	
Current	34 (37.0%)	14 (17.7%)	5 (7.9%)	
Smoking (pack-years), IQR	15.0 (0.0–32.5)	20.0 (0.0–40.0)	20.0 (10.0–40.0)	
**Comorbidity**				
Diabetes mellitus	24 (26.1%)	26 (32.9%)	12 (19.0%)	0.176
COPD	7 (7.6%)	7 (8.9%)	10 (15.9%)	0.220
GERD	27 (29.3%)	27 (34.2%)	19 (30.2%)	0.777
Asthma	5 (5.4%)	5 (6.3%)	6 (9.5%)	0.598
Old pulmonary tuberculosis	18 (19.6%)	15 (19.0%)	13 (20.6%)	0.970
Cancer	23 (25.0%)	15 (19.0%)	14 (22.2%)	0.641
Coronary artery disease	19 (20.9%)	12 (15.2%)	13 (20.6%)	0.587
Cerebrovascular disease	5 (5.4%)	2 (2.5%)	2 (3.2%)	0.585
**Pulmonary function test (at IPF diagnosis)**				
FVC (L)	2.8 ± 0.9	2.5 ± 0.7	2.8 ± 0.6	0.582
FVC % pred	83.6 ± 20.0	77.6 ± 12.9	76.3 ± 13.1	0.004
FEV_1_ (L)	2.2 ± 0.7	2.0 ± 0.5	2.2 ± 0.4	0.586
FEV_1_% pred	96.2 ± 22.6	93.3 ± 16.1	87.6 ± 12.9	0.005
DL_CO_ (mL/mmHg/min)	13.0 ± 4.6	10.3 ± 3.5	12.0 ± 3.3	0.126
DL_CO_ % pred	69.8 ± 25.5	62.8 ± 16.4	65.0 ± 15.1	0.103
**Severity of IPF (GAP stage)**				0.230
I	68 (73.9%)	50 (63.3%)	43 (68.3%)	
II	21 (22.8%)	28 (35.4%)	20 (31.7%)	
III	3 (3.3%)	1 (1.3%)	0 (0.0%)	
Follow-up period (months), IQR	31.1 (12.6–54.8)	24.4 (14.2–32.7)	24.1 (15.2–31.2)	0.072

Values are expressed as the mean ± standard deviation, number (%) or mean (interquartile range).

*BMI* body mass index, *COPD* chronic obstructive pulmonary disease, *DL*_*CO*_ diffusing capacity of the lungs for carbon monoxide, *FEV*_*1*_ forced expiratory volume in one second, *FVC* forced vital capacity, *GAP* gender, age, and physiology, *GERD* gastroesophageal reflux disease, *IPF* idiopathic pulmonary fibrosis, *IQR* interquartile range.

### Medication dose distribution

**Figure 1:**
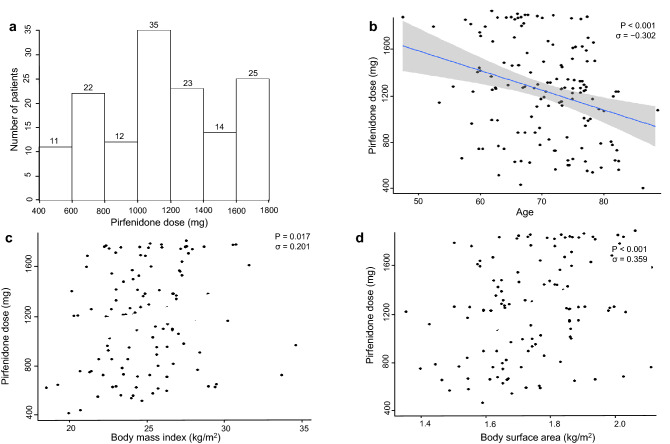
(**a**) Distribution of pirfenidone dose (mg/day). (**b**–**d**) Scatter plot showing the relationship among age, body mass index, body surface area, and pirfenidone dose. Age and pirfenidone dose were negatively correlated (Pearson’s correlation efficiency, σ = − 0.302, p < 0.001). Body mass index, body surface area, and pirfenidone dose were positively correlated (Pearson's correlation efficiency, σ = 0.201, p = 0.017; Pearson's correlation efficiency, σ = 0.359, p < 0.001, respectively).

The distribution of pirfenidone dose per day is shown in Fig. 1a. The median of pirfenidone dose was 1185 (IQR, 847–1456) mg. As shown in Table 1, patients in the high-dose group were younger and with higher BMI and BSA. To confirm the correlation among age, BMI, BSA, and medication dose, Pearson’s correlation test was performed. Age and medication dose showed a significant negative correlation (σ = − 0.302, p < 0.001, Fig. 1b). BMI, BSA, and medication dose showed a significant positive correlation (σ = 0.201, p = 0.017, Fig. 1c; σ = 0.3591, p < 0.001, Fig. 1d, respectively). This finding suggests that patients with younger age, and higher BMI and BSA tolerated higher medication dose.

### Treatment effect

**Figure 2:**
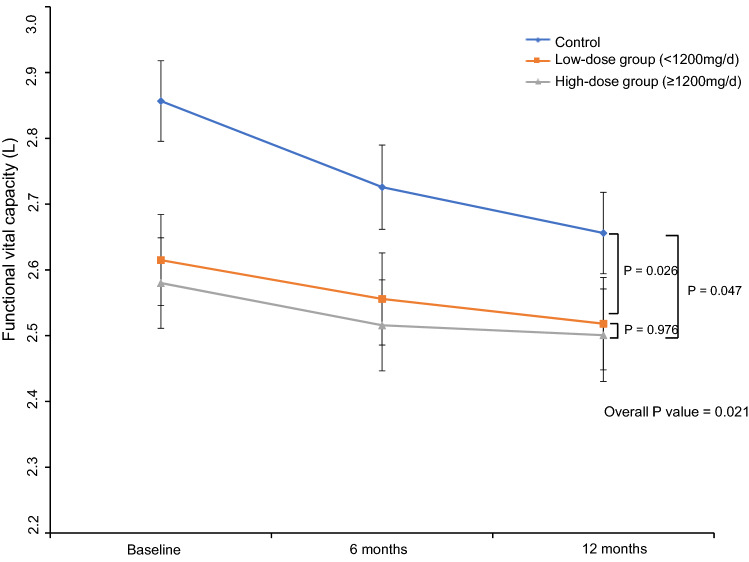
FVC changes according to pirfenidone dose. The FVC declined more significantly in the control group compared with the high-dose and low-dose groups (overall p = 0.021). There was no significant difference in FVC change between the low-dose and high-dose groups (p = 0.976). *FVC* Forced vital capacity.

As sex, age, height, and weight, which can affect the FVC value, were different among the groups, we adjusted these four variables and evaluated the changes in the FVC value among the groups. The adjusted mean changes in FVC in the first year were − 200.7 ± 28.2, − 88.4 ± 31.4, and − 94.7 ± 35.3 mL in the control, low-dose, and high-dose groups, respectively. Overall, the FVC change among the groups showed a significant difference (p = 0.021, Fig. 2). The FVC declined more significantly in the control group than in the high-dose group, with an adjusted mean FVC change difference of 106.0 ± 45.2 mL (p = 0.047). The FVC also declined more significantly in the control group than in the low-dose group, with an adjusted mean FVC change difference of 112.4 ± 42.2 mL (p = 0.026). There was no significant difference in the FVC change between the low-dose and high-dose groups (p = 0.976). The adjusted mean FVC changes among the groups are shown in Fig. 2. The FVC change in the second year was − 76.6 ± 10.8 and − 63.5 ± 44.8 mL in the low-dose and high-dose groups, respectively, indicating that the effect of low-dose pirfenidone persisted in the second year (Supplementary Fig. 1).

### BSA-adjusted pirfenidone dosing

BSA-adjusted pirfenidone dose distribution is shown in Supplementary Fig. 2. The BSA-adjusted pirfenidone dose was normally distributed based on the mean 665.0 ± 201.8 mg/m^2^ (Shapiro–Wilk normality test = 0.108, Supplementary Fig. 2). When the difference in FVC decline was evaluated by BSA-adjusted pirfenidone dose based on the mean value, the low (< 665.0 mg/m^2^) and high BSA-adjusted pirfenidone groups (≥ 665.0 mg/m^2^) did not show significant difference in FVC change. In contrast, the low and high BSA-adjusted pirfenidone groups showed a decreasing trend in FVC change compared with the control group, although the difference was not statistically significant (p = 0.051). The adjusted mean FVC changes among the groups according to BSA-adjusted pirfenidone dose are shown in Supplementary Fig. 2b. The results suggest that even when the pirfenidone dose is adjusted by BSA, the low-dose group has the same effect as the high-dose group in reducing FVC decline compared with the control group.

### Adverse events

The AEs that occurred during the study period in the pirfenidone treatment group are shown in Table 2. In 142 patients who were treated with pirfenidone, the majority of AEs were related to the GI tract or skin. The most frequent AEs were dyspepsia (38.7%), anorexia (25.4%), and rash (25.4%). Elevations in the level of alanine or aspartate aminotransferase (values that were three or more times the upper limit of the normal range) were observed in 8 (5.6%) patients. A comparison the frequency of AEs according to the dose revealed that dyspepsia (48.1% vs. 27.0%, p = 0.017), anorexia (35.4% vs. 12.7%, p = 0.004), and nausea (17.7% vs. 1.6%, p = 0.005) were significantly more frequent in the low-dose group than in the high-dose group. Skin-related adverse events did not show a difference between the low-dose and high-dose groups. These findings suggest that dose reduction is mainly attributed to GI tract-related AEs. Furthermore, the fact that patients with younger age and higher BMI and BSA showed more tolerance to higher medication doses suggests that they could be more tolerable to GI tract-related AEs.

**Table 2:** Adverse events according to pirfenidone dose.

	Total (n = 142)	Low-dose (n = 79)	High-dose (n = 63)	P value
Dyspepsia	55 (38.7%)	38 (48.1%)	17 (27.0%)	0.017
Anorexia	36 (25.4%)	28 (35.4%)	8 (12.7%)	0.004
Rash	36 (25.4%)	23 (29.1%)	13 (20.6%)	0.337
Fatigue	23(16.2%)	11 (13.9%)	12 (19.0%)	0.552
Photosensitivity	16 (11.3%)	8 (10.1%)	8 (12.7%)	0.830
Nausea	15 (10.6%)	14 (17.7%)	1 (1.6%)	0.005
Dizziness	10 (7.0%)	7 (8.9%)	3 (4.8%)	0.536
Weight loss	8 (5.6%)	5 (6.3%)	3 (4.8%)	0.971
GERD	8 (5.6%)	4 (5.1%)	4 (6.3%)	1.000
Liver enzyme elevation	8 (5.6%)	5 (6.3%)	3 (4.8%)	0.971
Diarrhea	5 (3.5%)	1 (1.3%)	4 (6.3%)	0.240
Insomnia	7 (4.9%)	6 (7.6%)	1 (1.6%)	0.210
Headache	4 (2.8%)	2 (2.5%)	2 (3.2%)	1.000
Constipation	1(0.7%)	0 (0.0%)	1 (1.6%)	0.909

Values are expressed as number (%).

*GERD* gastroesophageal reflux disease.

### Acute exacerbation

Among the 234 patients with IPF, 36 patients experienced acute exacerbation during the study period. Furthermore, 10 (10.9%), 11 (14.1%), and 15 (23.8%) patients experienced acute exacerbation in the control, low-dose, and high-dose groups, respectively. No statistically significant difference was observed among the groups.

## Discussion

Here, we evaluated the efficacy of low-dose pirfenidone (< 1200 mg/day) on disease progression based on FVC change and found that both low-dose and high-dose groups have similar efficacy in FVC decline compared with the control group. Dose reduction of pirfenidone is mainly attributable to GI tract-related AEs, and patients with younger age and higher BMI and BSA were treated with higher doses.

Although pirfenidone is reported to be a safe and tolerable drug^14^, a considerable number of patients discontinue or reduce the medication dose due to AEs. Adverse event-related discontinuation has been reported to be 14.4–15.0% in clinical trials and 15.3–24.3% in actual clinical practice^11–13,15^. Regarding dose reduction, a Japanese post marketing surveillance of pirfenidone reported that 61.8% patients received less than 1200 mg/day pirfenidone most frequently during the treatment period, although the approved dose is 1800 mg/day, which is lower than that in the United States and European countries^13^. A pooled analysis of three pivotal phase III trials, CAPACITY (Study 004 and Study 006)^9^ and ASCEND (Study 016)^10^ trials, which evaluated pirfenidone in IPF patients, revealed that, among 623 patients who were intended to treat with pirfenidone 2403 mg/day, 59.7% patients had temporary dose reduction and 31.5% had permanent dose reduction^16^. Despite discontinuation and dose modification in such a large proportion of patients, it is not clear whether the efficacy in these patients is similar to that in patients taking full doses.

In a pooled analysis of phase III trials, to evaluate changes in efficacy following dose modification, the patients were divided based on dose intensity > 90% and ≤ 90%. The study reported that both groups of patients showed a significantly smaller decrease in the annual rate of FVC decline than the placebo group^16^. In the study, the dose intensity cutoff was relatively high at 90%; therefore, it was difficult to obtain information on whether pirfenidone would be effective for patients who take lower doses in actual clinical settings.

A Japanese phase III clinical trial has shown that patients taking 1200 mg/day pirfenidone presented a similar efficacy in reducing FVC decline as patients taking 1800 mg/day pirfenidone^8^. Therefore, we evaluated whether a lower dose (< 1200 mg/day) of pirfenidone would be as effective as a dose higher than 1200 mg pirfenidone and found that the lower dose was similarly effective as the high dose compared with the control group. We additionally evaluated the efficacy of pirfenidone at a dose of less than 1000 mg and found that it has a significant effect in reducing FVC decline (Supplementary Fig. 3). Unlike previous studies that evaluated medication dose as daily dose administered most frequently during the treatment period^13,16,17^, in the present study, the medication dose was analyzed using the average dose that the patients took during the treatment period, considering actual clinical settings in which the dose is reduced and re-increased according to patient’s conditions and AEs. We believe that using the average dose to evaluate the efficacy is more reasonable than using the most frequently administered dose, which is a strength of this study.

We also evaluated the efficacy of pirfenidone according to the BSA-adjusted dose based on the mean value of 665.0 mg/m^2^ and revealed that the high-dose and low-dose groups showed a similar reduced FVC decline trend. A previous retrospective study in Japan evaluated the efficacy of pirfenidone according to the BSA-adjusted dose and reported that, based on the median value of BSA-adjusted pirfenidone dose in their study population (876 mg/m^2^), patients receiving a higher dose showed significantly lower annual decline FVC than those taking lower doses. The study suggested that pirfenidone dosing regimen based on the BSA-adjusted dose might be useful^17^. However, the study population was too small (n = 23) to be generalized. Regarding dosing of pirfenidone based on the BSA-adjusted dose, further studies are required to evaluate its usefulness in efficacy and treatment-related AEs.

Our study had some limitations. First, we used a retrospective nonrandomized design, which is associated with various biases and confounding factors. Second, approximately 60% of the patients who did not follow up or were transferred to other hospitals were excluded. This selection bias may have influenced the final result. Third, this study was conducted in Korea, and therefore, the results cannot be directly applied to European and North American populations that take pirfenidone 2400 mg as a full dose. Finally, the information on the plasma concentration of the metabolite of the drug was not available to confirm the dose-dependent effects.

In conclusion, the present study revealed that low-dose pirfenidone (< 1200 mg/day) is also effective in reducing FVC decline. Dose reduction may help patients to better control AEs, especially GI tract-related AEs. We expect that providing clear instructions to physicians for dose modification to control AEs and educating patients to continue the medication even at low doses can help reduce FVC decline based on our study result, that is, taking pirfenidone at low doses is also effective.

## Methods

### Patients

**Figure 3:**
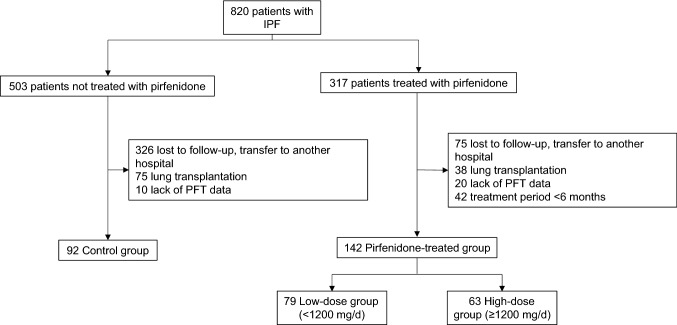
Patient recruitment flow chart. *IPF* idiopathic pulmonary fibrosis, *PFT* pulmonary function test.

We retrospectively reviewed the medical records of patients with IPF who were treated in the Severance Hospital, a tertiary care university hospital in South Korea, between January 2013 and March 2018. The inclusion criteria were as follows: patients (1) diagnosed with IPF, (2) followed up for more than one year, and (3) who underwent pulmonary function test for more than two times. Eight hundred and twenty patients with IPF were identified, and among those patients, 317 were treated with pirfenidone at least once and 503 patients were not treated with pirfenidone. Five hundred and eighty-six patients were excluded for the following reasons (Fig. 3): follow-up loss (n = 401), incomplete pulmonary function test results (n = 30), lung transplantation (n = 113), and had taken pirfenidone for less than 6 months (n = 42). Finally, 142 patients who were treated with pirfenidone for more than 6 months and 92 patients who were not treated with pirfenidone were included in the study. This study was approved by the Institutional Review Board and Ethics Committee of Severance Hospital (IRB number: 4-2018-0435). All methods were performed in accordance with the Declaration of Helsinki. Written informed consent was waived as the nature of retrospective study by IRB.

### Definitions

Idiopathic pulmonary fibrosis was diagnosed via a multidisciplinary approach involving pulmonologists as well as radiologists and pathologists specializing in chest diseases, in accordance with the diagnostic criteria set by the International Consensus Statement of the American Thoracic Society and European Respiratory Society in 2011^3^. The gender–age–physiology (GAP) index was applied to assess the severity of IPF. The GAP index was calculated using gender (0–1 point), age (0–2 points), FVC (0–2 points), and diffusing capacity of the lungs for carbon monoxide (DL_CO_; 0–3 points), and categorized into the following three stages: I (0–3 points), II (4–5 points), and III (6–8 points)^18^. Acute exacerbation of IPF (AE-IPF) was defined as acute, clinically significant respiratory deterioration characterized by evidence of new widespread alveolar abnormality; this meets the diagnostic criteria proposed by Collard et al.^19^.

### Pirfenidone dose

Pirfenidone was initially prescribed at 600 mg/day dose, and the dose was increased every 1–2 weeks until the total dose reached 1800 mg/day, unless the patients experienced severe adverse event (AE) and therefore could not continue pirfenidone. Studies have reported the gastrointestinal (GI) tract-related (e.g., nausea, dyspepsia, and diarrhea) and skin-related (e.g., photosensitivity and rash) AEs occur at a high frequency^5,11,12,20^. These AEs can be alleviated by adjuvant treatments such as proton pump inhibitors, broad-spectrum sunscreen or topical steroids, and dose reduction or interruption^20^. For these reasons, it is often observed that dose reduction or interruption is necessary, depending on the severity of the AE, rather than maintaining a constant dose in actual clinical practice. Therefore, in the present study, we defined the dose of pirfenidone per day as “total amount of pirfenidone prescribed from the initiation of the drug divided by the observation period,” which represents the average dose the patients took during the treatment period. Additionally, to evaluate the efficacy of pirfenidone dose adjusted according to the body size, the average dose of pirfenidone was divided by body surface area (BSA), which was calculated using the Du Bois formula^21^.

### Statistical analysis

The changes in FVC from the baseline to 12 months were assessed using the linear mixed model with Bonferroni correction. Age, sex, height, and weight that affect the FVC value were adjusted in the linear mixed model. Continuous variables were analyzed using the analysis of variance and Kruskal–Wallis test. Categorical variables were analyzed using chi-squared distribution and Fisher’s exact tests. In all cases, the results with a p value of < 0.05 were considered statistically significant. All statistical analyses were performed using SAS program, version 9.4 (SAS Institute, Cary, NC, USA).

### Ethics approval and consent to participate

This study was approved by the Institutional Review Board and Ethics Committee of Severance Hospital (IRB number: 4-2018-0435). Written informed consent was waived as the nature of retrospective study by IRB.

## Supplementary information

Supplementary Legends.

Supplementary Figures.

## Data Availability

The datasets used and analyzed in the current study are available from the corresponding author on reasonable request.

## Abbreviations

AE: Adverse event
AE-IPF: Acute exacerbation of IPF
BMI: Body mass index
BSA: Body surface area
DL_CO_: Diffusing capacity of the lungs for carbon monoxide
FEV_1_: Forced expiratory volume in one second %
FVC: Forced vital capacity
GAP: Gender–age–physiology
GI: Gastrointestinal
IPF: Idiopathic pulmonary fibrosis
IQR: Interquartile range
