# Supplementary material for: Efficacy of low dose pirfenidone in idiopathic pulmonary fibrosis: real world experience from a tertiary university hospital
Source: Sci Rep. 2020 Dec 4;10:21218. doi: 10.1038/s41598-020-77837-x (PMC7719184; doi:10.1038/s41598-020-77837-x)
Supplement: Supplementary file 1 — Supplementary Legends. [file 41598_2020_77837_MOESM1_ESM.docx]

**Efficacy of low dose pirfenidone in idiopathic pulmonary fibrosis: Real world experience from a tertiary university hospital**

Myung Jin Song^1^, Sung Woo Moon^2^, Ji Soo Choi^2^, Sang Hoon Lee^2^, Su Hwan Lee^2^, Kyung Soo Chung^2^, Ji Ye Jung^2^, Young Ae Kang^2^, Moo Suk Park^2^, Young Sam Kim^2^, Joon Chang^2^ and Song Yee Kim^2, *^

^1^Division of Pulmonary and Critical Care Medicine, Department of Internal Medicine, Seoul National University College of Medicine, Seoul National University Bundang Hospital, Seongnam, South Korea

^2^Division of Pulmonology, Department of Internal Medicine, Severance Hospital, Yonsei University College of Medicine, Seoul, Republic of Korea

**^*^Corresponding author**

Song Yee Kim, MD, PhD

Division of Pulmonology, Department of Internal Medicine, Severance Hospital, Yonsei University College of Medicine, 50-1 Yonsei-ro, Seodaemun-gu, Seoul 03722, Republic of Korea

E-mail address: dobie@yuhs.ac

Supplementary figure 1. Changes in FVC according to the pirfenidone dose up to the second year.

FVC, Forced vital capacity

Supplementary figure 2. (**a**) Distribution of BSA-adjusted pirfenidone dose (mg/m^2^). (**b**) FVC changed according to BSA-adjusted pirfenidone dose. The FVC change tended to decrease in the low-dose and high-dose pirfenidone groups compare with the control group (p = 0.051).

BSA, body surface area; FVC, Forced vital capacity

Supplementary figure 3. Changes in FVC according to the pirfenidone dose (control, <1000 mg/d, ≥1000 mg/d)

FVC, Forced vital capacity
