# Supplementary material for: Efficacy of low dose pirfenidone in idiopathic pulmonary fibrosis: real world experience from a tertiary university hospital
Source: Sci Rep. 2020 Dec 4;10:21218. doi: 10.1038/s41598-020-77837-x (PMC7719184; doi:10.1038/s41598-020-77837-x)
Supplement: Supplementary file 2 — Supplementary Figures. [file 41598_2020_77837_MOESM2_ESM.pptx]

## Slide 1
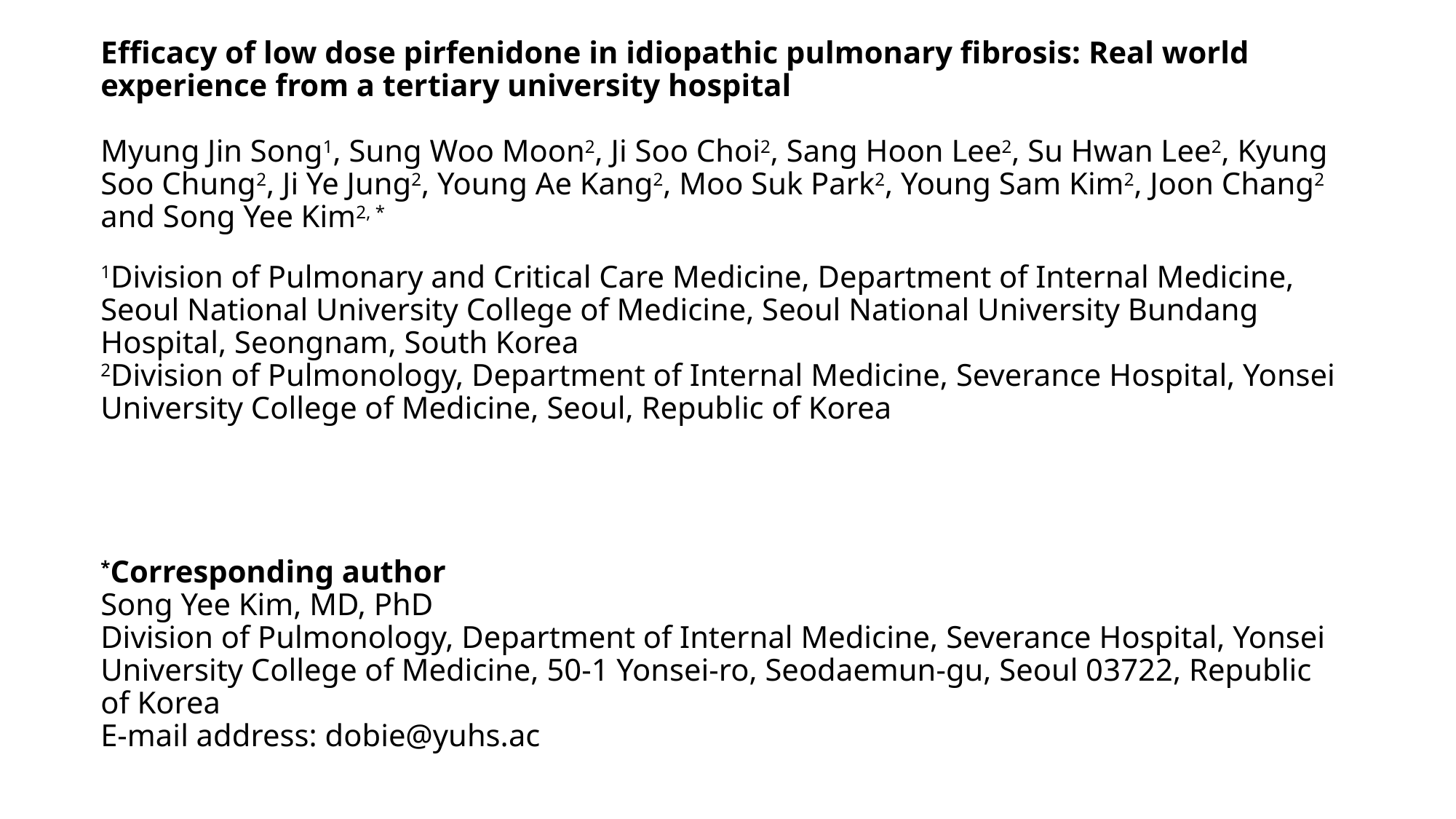

# Efficacy of low dose pirfenidone in idiopathic pulmonary fibrosis: Real world experience from a tertiary university hospital   Myung Jin Song1, Sung Woo Moon2, Ji Soo Choi2, Sang Hoon Lee2, Su Hwan Lee2, Kyung Soo Chung2, Ji Ye Jung2, Young Ae Kang2, Moo Suk Park2, Young Sam Kim2, Joon Chang2 and Song Yee Kim2, *   1Division of Pulmonary and Critical Care Medicine, Department of Internal Medicine, Seoul National University College of Medicine, Seoul National University Bundang Hospital, Seongnam, South Korea 2Division of Pulmonology, Department of Internal Medicine, Severance Hospital, Yonsei University College of Medicine, Seoul, Republic of Korea   *Corresponding author Song Yee Kim, MD, PhD Division of Pulmonology, Department of Internal Medicine, Severance Hospital, Yonsei University College of Medicine, 50-1 Yonsei-ro, Seodaemun-gu, Seoul 03722, Republic of Korea E-mail address: dobie@yuhs.ac

## Slide 2
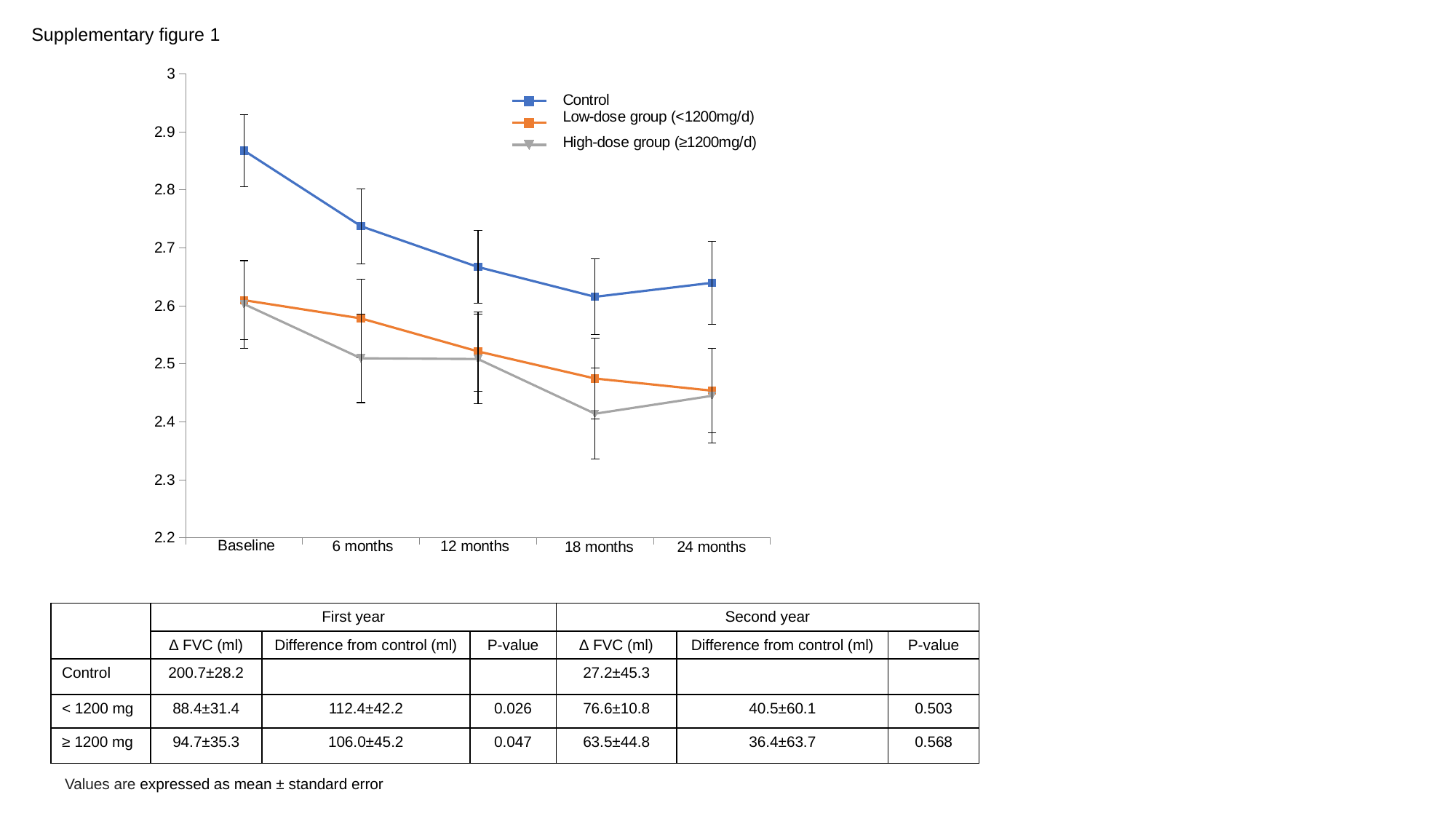

Supplementary figure 1
### Chart
| Category | control | pirfenidone < 1200 | pirfenidone ≥ 1200 |
|---|---|---|---|| | First year | | | Second year | | |
| --- | --- | --- | --- | --- | --- | --- |
| | ∆ FVC (ml) | Difference from control (ml) | P-value | ∆ FVC (ml) | Difference from control (ml) | P-value |
| Control | 200.7±28.2 | | | 27.2±45.3 | | |
| < 1200 mg | 88.4±31.4 | 112.4±42.2 | 0.026 | 76.6±10.8 | 40.5±60.1 | 0.503 |
| ≥ 1200 mg | 94.7±35.3 | 106.0±45.2 | 0.047 | 63.5±44.8 | 36.4±63.7 | 0.568 |
Values are expressed as mean ± standard error

## Slide 3
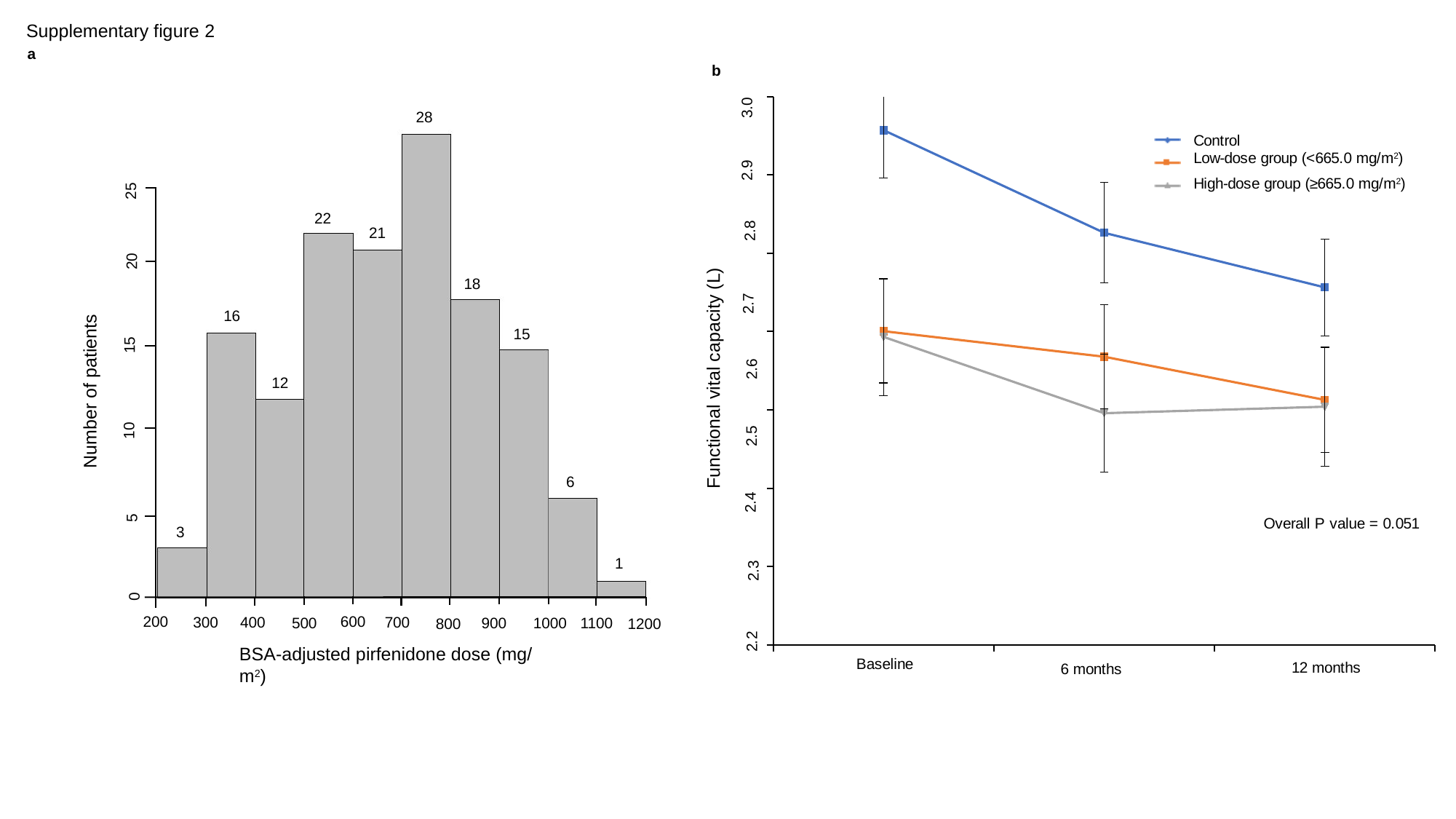

Supplementary figure 2
a
b
### Chart
| Category | control | pirfenidone < 1200 | pirfenidone ≥ 1200 |
|---|---|---|---|
| 1 | 2.8571 | 2.6005 | 2.5932 |
| 2 | 2.7261 | 2.5679 | 2.4956999999999994 |
| 3 | 2.6563999999999997 | 2.5126999999999997 | 2.504 |Functional vital capacity (L)
28
25
22
21
20
18
16
15
15
12
10
6
5
3
1
0
600
200
300
400
700
500
900
1000
1100
1200
800
BSA-adjusted pirfenidone dose (mg/m2)
Number of patients

## Slide 4
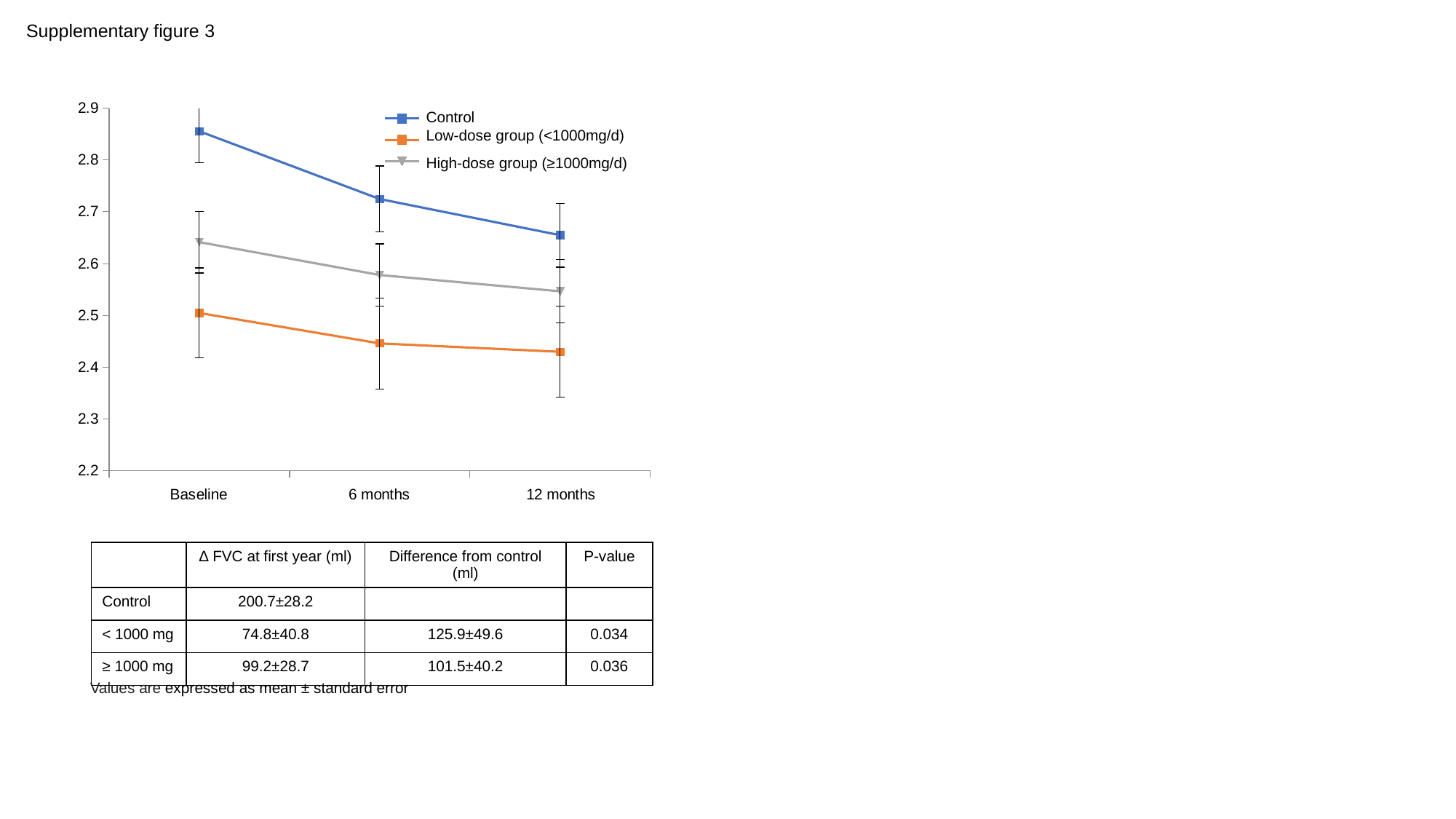

Supplementary figure 3
### Chart
| Category | control | pirfenidone < 1000 | pirfenidone ≥ 1000 |
|---|---|---|---|Control
Low-dose group (<1000mg/d)
High-dose group (≥1000mg/d)
| | ∆ FVC at first year (ml) | Difference from control (ml) | P-value |
| --- | --- | --- | --- |
| Control | 200.7±28.2 | | |
| < 1000 mg | 74.8±40.8 | 125.9±49.6 | 0.034 |
| ≥ 1000 mg | 99.2±28.7 | 101.5±40.2 | 0.036 |
Values are expressed as mean ± standard error
